# Demonstration of conventional soliton, bound-state soliton, and noise-like pulse based on chromium sulfide as saturable absorber

**DOI:** 10.1515/nanoph-2022-0483

**Published:** 2022-11-03

**Authors:** Fuhao Yang, Zhiqi Sui, Shuo Sun, Si Chen, Yanjuan Wang, Weiyu Fan, Shuaimeng Li, Guomei Wang, Wenfei Zhang, Cheng Lu, Shenggui Fu, Huanian Zhang

**Affiliations:** School of Physics and Optoelectronic Engineering, Shandong University of Technology, Zibo 255049, China; School of Physics and Electronics, Shandong Normal University, Jinan 250358, China

**Keywords:** bound-state soliton, chromium sulfide, conventional soliton, noise-like pulse, saturable absorber

## Abstract

Ferromagnetic semiconductor chromium sulfide (Cr_2_S_3_), as a member of transition metal chalcogenide (TMC), exhibits the narrow bandgap value of 0.45 eV theoretically and has been applied in photoelectric field. However, the application in ultrafast fiber laser of Cr_2_S_3_ has not been investigation at present. In this work, the Cr_2_S_3_-based SA was successfully prepared by depositing nanosheets onto tapered fiber. The conventional soliton (CS) operation, three pulse bound-state (BS) soliton operation, and noise-like pulse (NLP) operation around 1531 nm are observed from 80 mW to 147 mW in an EDFL. The experimental results demonstrated that Cr_2_S_3_ as a promising 2D material has tremendous potential in designing ultrafast photonics device.

## 1 Introduction

Ultrafast fiber laser with a pulse width of picosecond or femtosecond range has aroused more focus from researchers in the field of optical communication, biomedical, material processing, and so on [1–4]. Due to advantage of simple structure and economy, passive mode-locked technique is a common way to soon realize mode-locked pulse in ultrafast fiber lasers [5–7]. In addition, the key element for implementing this technique in passively mode-locked fiber lasers is the stable and reliable saturable absorber (SA), and the category of SA could be divided into artificial SAs and real SAs. The conventional artificial SAs are vulnerable to environment, which output power is relatively low and difficult to self-start, such as nonlinear polarization rotation (NPR), nonlinear polarization evolution (NPE), and nonlinear amplifying loop mirror (NALM). However, with the wide application of polarization-maintaining (PM) fiber and the introduction of phase bias technique, the resistance to interference from external environment and self-starting ability with high output power were enhanced in fiber laser [8–11]. In addition, the real SAs are also quickly spread across the world and considered as high-efficient methods to obtain mode-locked or Q-switched operation. In recent years, numerous SA materials with extraordinary nonlinear absorption characteristics experienced rapid evolution from semiconductor materials to nanomaterials [12–24]. Especially the emergence of two-dimensional (2D) nanomaterials, particularly graphene possesses properties of wide absorption range, low saturation intensity, ultra-fast recovery time, high nonlinear optical absorption coefficient, and so on, attracted much attention to investigating 2D nanomaterials [25–31]. However, the nature with zero bandgap and low optical absorption quotiety limited its development. At present, there are many other 2D nanomaterials have been demonstrated its outstanding performance in succession including topological insulators (TIs) [32–34], ferromagnetic insulators (FIs) [35, 36], transition metal chalcogenides (TMCs) [37–45], black phosphorus (BP) [46–49], MXenes [50–53], single element two-dimensional materials (Xenes) [54–56], and so on.

Recently, 2D ferromagnetic materials have attracted more attention from increasingly. Cr_2_X_2_Te_6_ (X = Ge, Si) as a kind of distinguished FIs was prepared into SA and saturable absorption properties were investigated in an erbium-doped fiber laser (EDFL) [35, 36]. Generation of ultrashort pulse has demonstrated that Cr_2_X_2_Te_6_ with ferromagnetic property possessed outstanding saturable absorption capacity and potential application in a fiber laser. Hence, the investigation of new ferromagnetic materials has a grand significance in development of ultrafast photonics. Ferromagnetic semiconductor chromium sulfide (Cr_2_S_3_) exhibits theoretically narrow bandgap of 0.45 eV, which may be considered as a prospective 2D nonlayered material in the application of optoelectronic devices [57]. In the meanwhile, Cr_2_S_3_ as a member of TMCs, which has been attracted tremendous attention from researchers all over the world for its distinguished nonlinear optical performance [58–60]. In 2019, He et al. firstly used vdW epitaxy techniques and chemical vapor deposition (CVD) method to obtain a unit-cell-thick stable Cr_2_S_3_ semiconductor [61]. Until 2020, Zhang et al. synthesized Cr_2_S_3_ which controllable thickness was unit-cell thickness of 1.85 nm by CVD with low cost and simple; further demonstrated that the bandgap value of Cr_2_S_3_ was only ∼0.15 eV; the synthesized Cr_2_S_3_ exhibited excellent air stability and high-performance of photodetector application under wavelengths of 520, 808, and 1550 nm [62]. And some studies have shown that the conductive behavior of Cr_2_S_3_ will change as nanosheet thickness increasing from p-type to ambipolar and then to n-type [63]. Compared with other 2D materials, the research progress indicates that Cr_2_S_3_ due to its unique optoelectronic properties has great potential for designing excellent optoelectronic devices. However, the performance of Cr_2_S_3_ has not been demonstrated in nonlinear optics and ultrafast laser applications at present.

Cr_2_S_3_ was chosen for designing the SA in order to demonstrate application in EDFL. In this work, the SA was successfully fabricated by depositing Cr_2_S_3_ powder onto a piece of tapered fiber and the nonlinear transmission characteristic is investigated at 1.5 μm. The conventional soliton (CS) operation, three pulses bound-state soliton (BS) operation and noise-like pulse (NLP) operation were obtained in an EDFL. CS operation was located at 1530.8 nm, which spectral full width of half maximum (FWHM) is ∼4 nm, the pulse duration was calculated at about 4.60 ps by measuring autocorrelation trace and fitting curve. BS operation with the spectral center wavelength of 1530.7 nm exhibited two different modulated periods of 1.07 and 3.20 nm which corresponds to pulse intervals of 2.1 ps and 7.4 ps, respectively; NLP mode-locked operation was also achieved with increasing pump power. Experimental results adequate proved that Cr_2_S_3_ with distinguished saturable absorption capacity shows tremendous potential for designing high-performance ultrafast photonics devices.

## 2 Preparation and characterization of SA based on Cr_2_S_3_

Due to Cr_2_S_3_ powder and water could produce the chemical reaction, and the liquid-phase exfoliation (LPE) method cannot be employed to prepare nanosheets in this experiment. Therefore, the SA was fabricated tentatively by a special method and the preparation process is as followed: Firstly, the Cr_2_S_3_ powder is ground in an agate grinder bowl for more than 4 h. Then, the Cr_2_S_3_ powder with grinding carefully was sprinkled on tapered area of a piece of tapered fiber, which the tapered fiber was fixed on a glass plate. Finally, the tapered fiber was placed at 24 °C for 48 h, which aimed that Cr_2_S_3_ nanosheets were preferably deposited onto tapered area. The SA was fabricated successfully through above prepared process.

**Figure 1:**
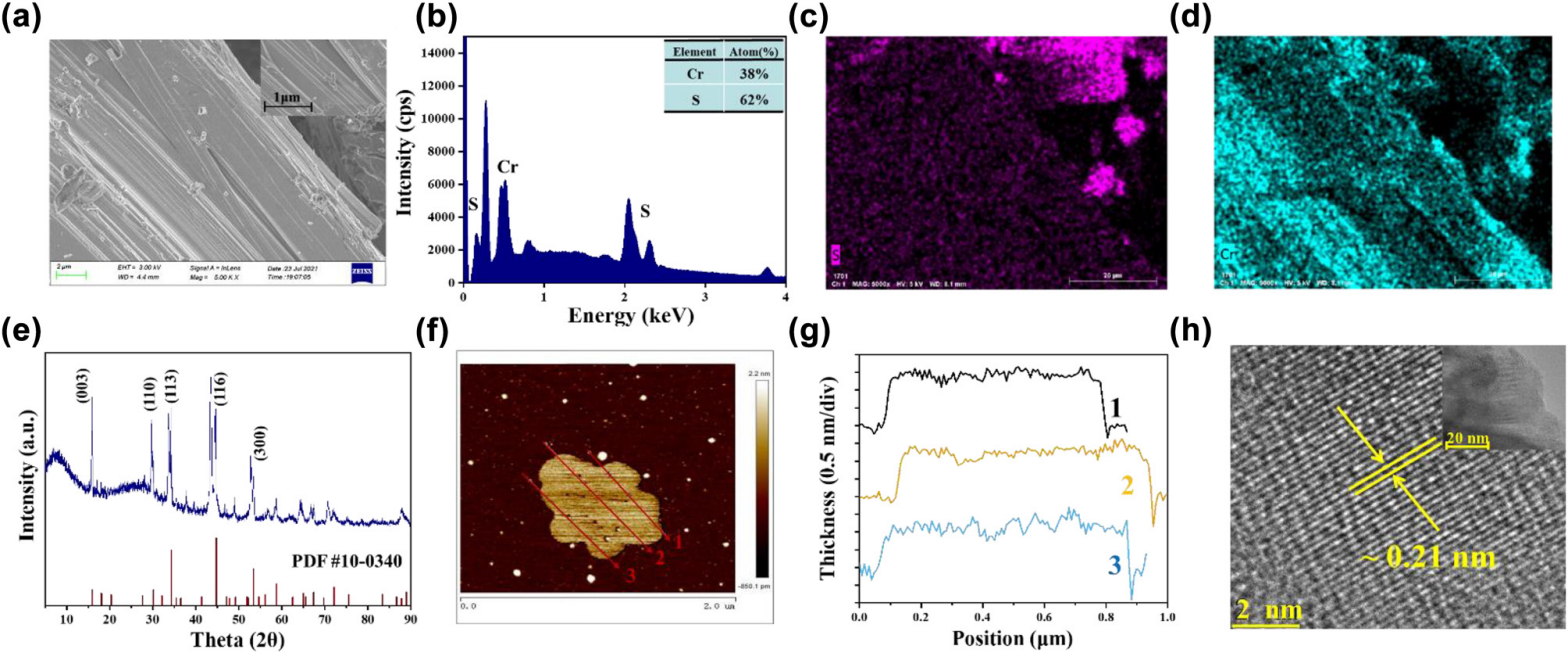
The characterization of Cr_2_S_3 _material. (a) SEM image of Cr_2_S_3_ power at resolution of 2 μm and inset of (a) SEM image at resolution of 1 μm. (b) EDS spectrum. (c) And (d) SEM-EDS mapping images. (e) XRD spectrum of Cr_2_S_3_ powder. (f) AFM image of prepared nanosheets. (g) The corresponding thickness characteristics. (h) HRTEM image with obvious crystal lattice. Inset is TEM images of 20 nm resolution.

The scanning electron microscope (SEM) image of Cr_2_S_3_ powder is shown in Figure 1(a) with resolution of 2 μm and the inset of Figure 1(a) is the image of 1 μm, which the surface morphology with an obvious layered structure of Cr_2_S_3_ powder is observed. The energy dispersion X-ray spectroscopy (EDS) is depicted in Figure 1(b), the typical peak of Cr and S could be clearly observed and the atom ratio of Cr and S is estimated at 38:62, corresponding to the chemical formula of Cr_2_S_3_. The SEM-EDS mapping images under resolution of 20 μm are shown in Figure 1(c) and (d), which shows that elements of Cr and S are uniform distribution. The X-ray diffraction (XRD) spectrum of Cr_2_S_3_ powder is shown in Figure 1(e) within 5° to 90°, distinct diffraction peaks are located at (003), (110), (113), (116), and (300), which could be matched well with the standard rhombohedral Cr_2_S_3_ pattern (PDF #10-0340). For the further detailed characterization of the layered structure characteristics of prepared Cr_2_S_3_ nanosheets, the atomic force microscope (AFM) and transmission electron microscope (TEM) are provided. As shown in Figure 1(f), the prepared nanosheets have uniform thickness and relatively flat surfaces. In addition, the nanosheet exhibits a thickness of 1.5 nm according to Figure 1(g). The high-resolution TEM image of resolutions 2 nm exhibits the lattice spacing of ∼0.2 nm as shown in Figure 1(h), which indicates that the prepared nanosheets have high crystallinity. The inset of Figure 1(h) is TEM image with resolution 20 nm, where shows the nanosheet has obvious crystal lattice structure.

**Figure 2:**
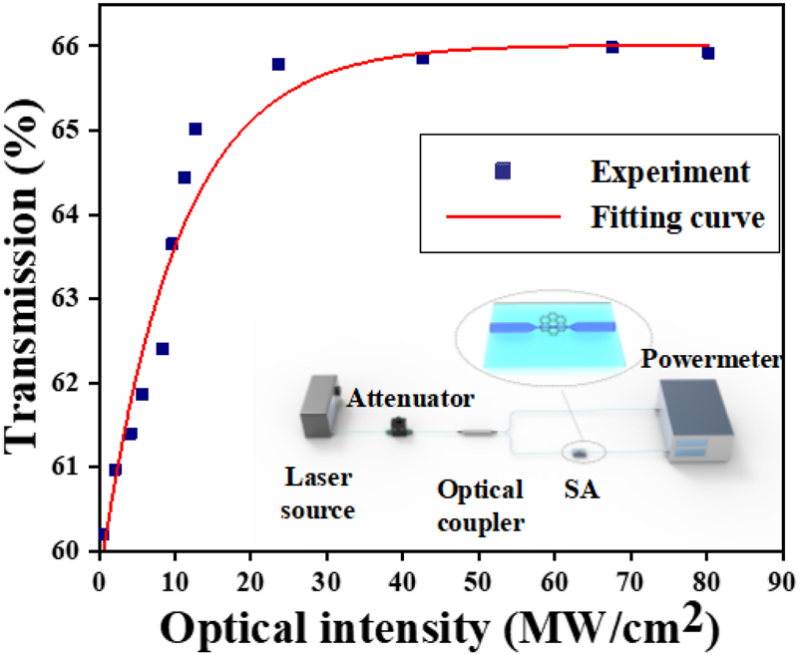
Nonlinear absorption curve of the Cr_2_S_3_-PVA film. (Inset) experimental setup of testing nonlinear optical property.

The nonlinear transmission characteristic based on Cr_2_S_3_ SA is investigated as shown in Figure 2, which employed a power-dependent transmission technique by a homemade femtosecond mode-locked fiber laser based on NPR technique (the central wavelength is 1562.28 nm, the fundamental frequency is 23.74 MHz, the pulse width is 464.47 fs) and the test setup is depicted in the inset of Figure 2. The data are fitted by the formula to obtain the fitting curve:

(1) $$T\left(I\right)=1-\Delta T\cdot \exp\left(-I/I_{\text{sat}}\right)-T_{\text{ns}}$$

where *T*(*I*) is the transmission rate, *T*_ns_ is nonsaturable loss, Δ*T* is modulation depth, *I* is input pulse energy, and *I*_sat_ is saturation energy, input pulse energy. Finally, the saturation intensity, modulation depth, and nonsaturable loss of the SA are calculated as 10.24 mW/cm^2^, 6.36%, and 65.01%, respectively.

## 3 Experimental setup

**Figure 3:**
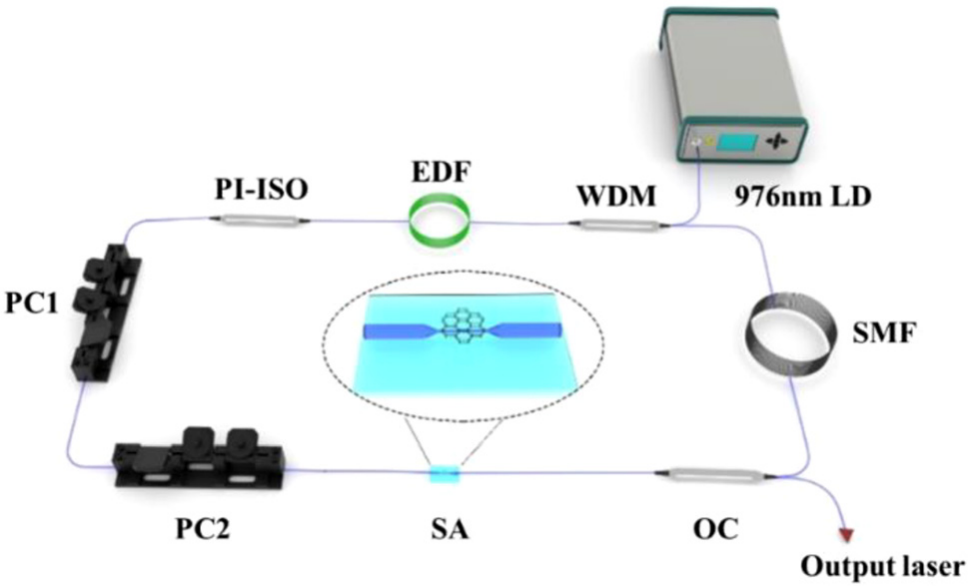
The experimental setup based on Cr_2_S_3_-SA EDFL.

The experimental setup is shown in Figure 3, pump source is a 980 nm laser diode (LD) and the pump light is delivered into ring cavity by a 980/1550 nm wavelength division multiplexer (WDM); the gain fiber selects 40 cm er-doped fiber (Er 80-4/125), which the dispersion parameter is −25 ps/nm/km; a polarization independent isolator (PI-ISO) is used to ensure unidirectional deliver of the laser in ring cavity. Two polarization controllers (PC1, PC2) are used to adjust the polarization state. SA based on Cr_2_S_3_ is placed between PC2 and output coupler (OC); The OC has coupling ratio of 90:10, which 10% output laser is used to detect. A piece of 10 m single-mode fiber (SMF) with the dispersion parameter of 17 ps/nm/km is employed for dispersion management. The total length of cavity is 13.94 m, the net dispersion in cavity of fiber laser is calculated at about −0.281 ps^2^. In addition, the output laser is detected by the digital oscilloscope (Wavesurfer 3054z), optical spectrum analyzer (Yokogawa AQ6370B), radio-frequency spectrum (Rohde&Schwarz FPC1000), and optical power meter.

## 4 Result and discussion

### 4.1 Conventional soliton operation in anomalous region

**Figure 4:**
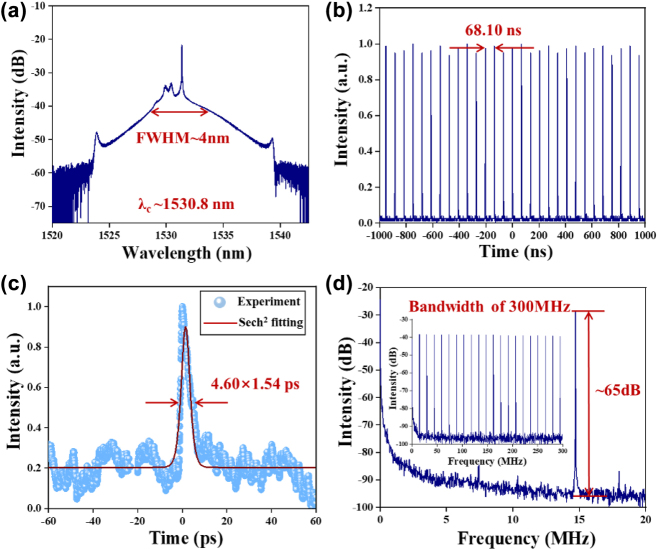
The output characteristics of Cr_2_S_3_-based SA conventional soliton mode-locked operation. (a) Emission optical spectrum. (b) Pulse train. (c) Measured and fitted autocorrelation trace. (d) RF spectrum is located at 14.74 MHz. (Inset of d) RF spectrum recorded within 300 MHz bandwidth.

In the experiment, when the pump power is higher than 80 mW, the stable mode-locked operation is observed by adjusting carefully the PCs. The mode-locked operation is maintained steadily from 80 mW to 147 mW of pump power, which the maximum output power reaches to 0.92 mW. At pump power from 80 to 90 mW, the CS mode-locked pulse is observed. Figure 4 shows the output characteristics of Cr_2_S_3_-based CS mode-locked operation. The emission optical spectrum is provided in Figure 4(a), Kelly sidebands are Symmetrically distributed on both sides of the emission optical spectrum, which the central wavelength is 1530.8 nm and the full width of half maximum (FWHM) is ∼4 nm. The top of CS spectrum has a slight continuous wave (CW) component, which is attributed to the birefringence effect of fiber and the nonlinear optical effect of SA. The pulse trains of the mode-locked operation are depicted in Figure 4(b), the pulse-to-pulse time is 68.10 ns corresponding to the fundamental frequency of 14.74 MHz, which matches accurately with the cavity length of 13.94 m. As shown in Figure 4(c), the autocorrelation trace of the mode-locked pulse is depicted, assuming a sech^2^ temporal profile calculation for CS operation, hence the real pulse duration is about 4.60 ps. According to the mentioned FWHM of emission optical spectrum with 4 nm, the time-bandwidth product (TBP) is about 2.36, which is larger than the theoretical transform limit value (0.315). The large TBP value indicates that the soliton pulse is a large frequency chirped due to the dispersion of cavity and fiber nonlinear effect. The RF spectrum is provided in Figure 4(d), the central frequency located at 14.74 MHz with a bandwidth of 20 MHz and the resolution of 100 Hz. The signal-to-noise ratio (SNR) is about 65 dB, which indicates that the mode-locked operation processes high stability. The RF spectrum within 300 MHz bandwidth is also depicted in the insert of Figure 4(d), which further reveals the mode-locked pulses have excellent stability.

### 4.2 The bound-state soliton mode-locked operation

**Figure 5:**
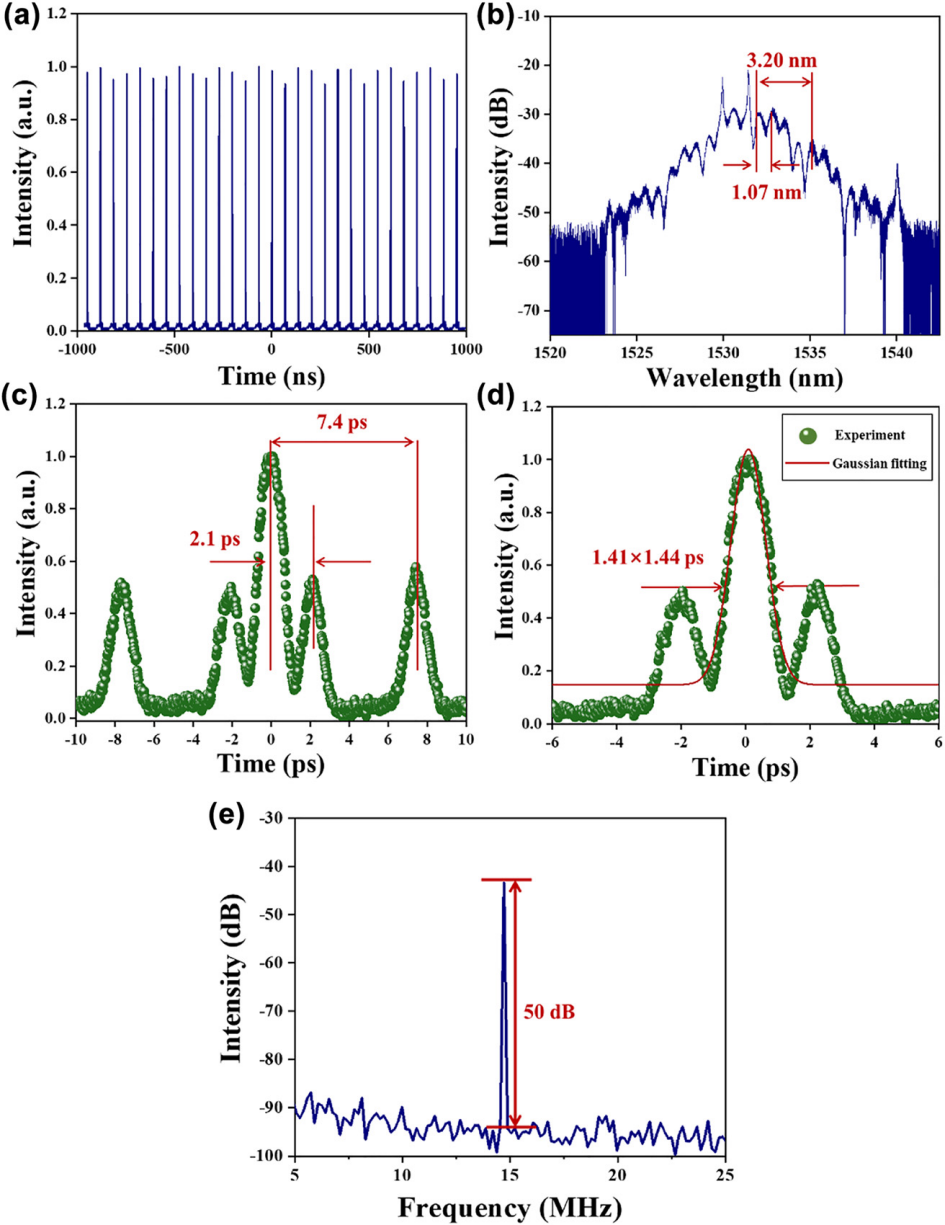
The output characteristics of Cr_2_S_3_-based SA bound-state soliton operation: (a) Pulse train. (b) Emission optical spectrum. (c) Autocorrelation trace of bound-state soliton. (d) The main-pulse fitting curve of bound-state soliton. (e) RF spectrum is located at 14.74 MHz.

When the pump power is higher than 90 mW, the mode-locked pulse starts to split slightly. Hence, the transition from CS mode-locked soliton to three-pulse bound-state soliton mode-locked is observed by regulating carefully the PCs, and the output characteristics at the pump power of 130 mW are depicted in Figure 5. The uniformly distributed bound-state soliton pulse sequence is provided in Figure 5(a) and the time interval of pulse is 67.82 ns. As shown in Figure 5(b), the optical spectrum of bound-state soliton located at central wavelength 1531.70 nm shows that the optical spectrum is symmetrical. There are two kinds of different pulse modulated periods with 1.07 nm and 3.20 nm in the optical spectrum of bound-state soliton, corresponding that the adjacent pulse has the different time separation and non-uniform distribution of peak position in Figure 5(c) and (d). Figure 5(c) shows that the autocorrelation trace of bound-state soliton mode-locked operation, there are five peaks with an intensity ratio of 1:1:2:1:1, which indicates that bound-state three soliton pulse has the same intensity and stable interval time of pulse-to-pulse. The separation time of main pulse to sub-pulse is 2.1 ps and 7.4 ps, respectively, corresponding to the spectral modulation period of 3.20 nm and 1.70 nm which only have a slight deviation compared with theoretical value. According to the Fourier transform, the spectral modulation Δ*λ* and the sub-pulse time interval Δ*T* satisfies the following relationship [64, 65]:

(2) $$\Delta T=\frac{\lambda_{0}^{2}}{\left(C\cdot \Delta \lambda_{0}\right)}$$

In this formula, *λ*_0_ is the central wavelength of optical spectrum, and *C* represents the speed of the light. Therefore, the modulation period of the spectrum with 3.20 nm and 1.70 nm corresponds to the time interval of 2.4 ps and 7.3 ps by formula, which is basically coincident with the observational result in Figure 5(c). The zoomed autocorrelation trace is shown in Figure 5(d), the real main-pulse duration is calculated at about 1.44 ps by Gaussian fitting. Figure 5(e) depicts the RF spectrum at BS mode-locked operation; the SNR reaches to 50 dB, which indicates the BS mode-locked operation works in a stable state.

### 4.3 Noise-like pulse operation

**Figure 6:**
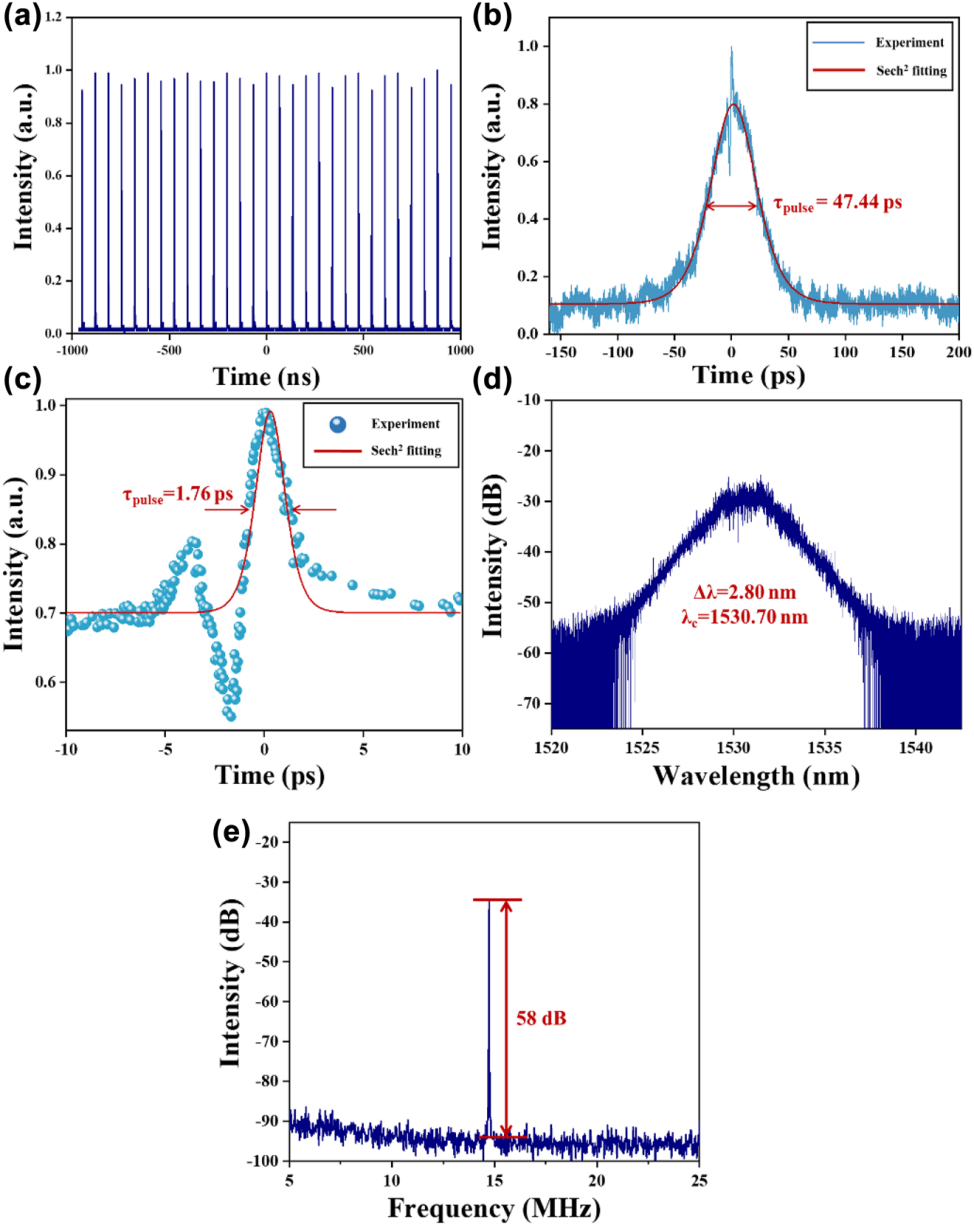
The output characteristics of the NLP operation based on Cr_2_S_3_-SA: (a) Pulse train. (b) Measured and fitting of autocorrelation trace of pulse. (c) Measured and fitting of autocorrelation trace of pulse spike. (d) The emission optical spectrum. (e) RF spectrum is located at 14.74 MHz.

**Figure 7:**
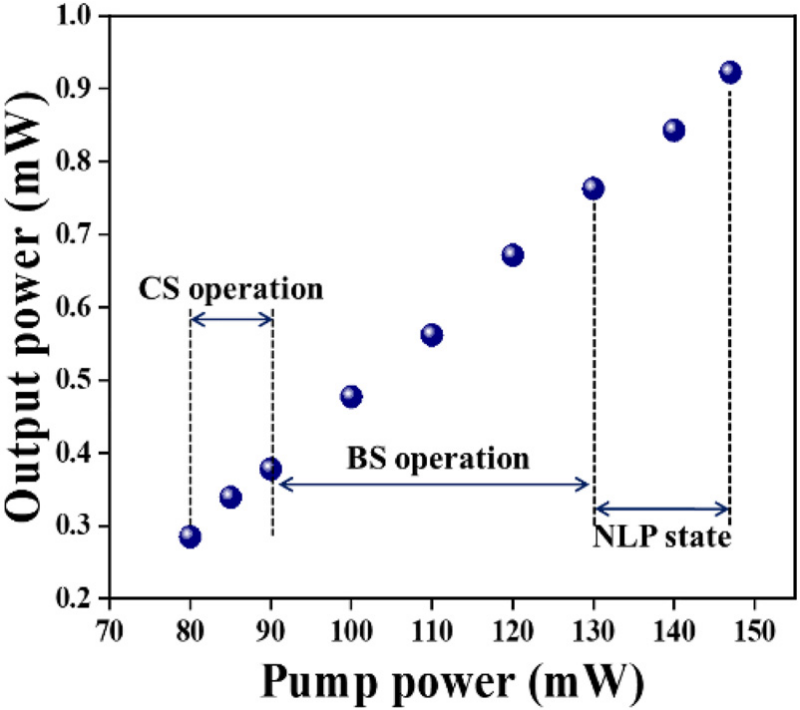
The relationship between pump power and output power at different mode-locked states.

When the pump power is more than 130 mW, the pulse splitting occurs again and BS soliton operation turns unstable. By adjusting the paddle of PCs and continuously increasing the pump power to 140 mW, the NLP mode-locked operation is observed, and stable mode-locked could be maintained from pump power of 130 mW–147 mW. Figure 6 presents the output characteristics of NLP under the pump power of 140 mW. Figure 6(a) shows the uniformly distributed oscilloscope pulse sequence of NLP operation, which the time interval is 67.83 ns and fundamental frequency is 14.74 MHz. The typical NLP operation of autocorrelation trace is depicted in Figure 6(b), which shows a spike ridding on the top of wide pedestal. The FWHM of pedestal is calculated at about 47.44 ps by sech^2^ fitting. As shown in Figure 6(c), the autocorrelation trace of pulse spike is presented and the FWHM of pulse trace is 1.76 ps by sech^2^ fitting. The emission optical spectrum of NLP also records in Figure 6(d), corresponding to the central wavelength of 1530.70 nm and the measured spectral FWHM of 2.80 nm. Those features are the typical characteristics of NLPs, which indicates that the soliton state is NLPs operation. As shown in Figure 6(e), the SNR of 58 dB indicates the NLP state is stable. Furthermore, the relationship of pump power and output power in different mode-locked operations is shown in Figure 7, in which tendency is an almost linear increasing from 80 to 147 mW.

## 5 Conclusions

In this experiment, the Cr_2_S_3_-SA is fabricated successfully by a novel method of depositing directly Cr_2_S_3_ powder onto a piece of tapered fiber. The saturable absorption performance is also investigated in an EDFL. When the pump power reaches to 80 mW, the stable CS mode-locked operation is observed by tuning polarization state. However, the CS soliton is limited by soliton area theory, when the pump power continues to be increased to 90 mW, the soliton is split into multi-soliton. And then, the split soliton is incorporated into a bound-state operation with fixed separation time by the direct soliton-to-soliton interaction [66]. When the pump power is increased to 130 mW, numerous ultrashort pulses randomly evolve into noise-like pulses, which is the combined effect of soliton collapse and positive feedback of cavity in the anomalous dispersion region [67]. The CS operation with a pulse width of 4.60 ps and spectral FWHM of 4 nm, three pulse BS operation, and NLP operation are successively obtained at central wavelengths of 1530.8 nm, 1531.70 nm, and 1530.7 nm, respectively. Their SNR of mode-locked pulse reaches to 65, 50, and 58 dB, indicating that mode-locked operation is stable. Our experimental result firstly demonstrates that the Cr_2_S_3_-SA possesses excellent saturable absorption properties, as well as is a promising optical modulator in ultrafast laser.
